# Origin of competing charge density waves in kagome metal ScV_6_Sn_6_

**DOI:** 10.1038/s41467-024-54702-3

**Published:** 2024-11-30

**Authors:** Kang Wang, Siyu Chen, Sun-Woo Kim, Bartomeu Monserrat

**Affiliations:** 1 Department of Materials Science and Metallurgy, University of Cambridge, Cambridge, UK; 2 Cavendish Laboratory, University of Cambridge, Cambridge, UK

**Keywords:** Electronic properties and materials, Phase transitions and critical phenomena

## Abstract

Understanding competing charge density wave (CDW) orders in the bilayer kagome metal ScV_6_Sn_6_ remains challenging. Experimentally, upon cooling, short-range order with wave vector ${{{{\bf{q}}}}}_{2}=(\frac{1}{3},\frac{1}{3},\frac{1}{2})$ forms, which is subsequently suppressed by the condensation of long-range ${{{{\bf{q}}}}}_{3}=(\frac{1}{3},\frac{1}{3},\frac{1}{3})$ CDW order at lower temperature. Theoretically, however, the **q**_2_ CDW is predicted as the ground state, leaving the CDW mechanism elusive. Here, using anharmonic phonon-phonon calculations combined with density functional theory, we predict a temperature-driven structural phase transitions from the high-temperature pristine phase to the **q**_2_ CDW, followed by the low-temperature **q**_3_ CDW, explaining experimental observations. We demonstrate that semi-core electron states stabilize the **q**_3_ CDW over the **q**_2_ CDW. Furthermore, we find that the out-of-plane lattice parameter controls the competing CDWs, motivating us to propose compressive bi-axial strain as an experimental protocol to stabilize the **q**_2_ CDW. Finally, we suggest Ge or Pb doping at the Sn site as another potential avenue to control CDW instabilities. Our work provides a full theory of CDWs in ScV_6_Sn_6_, rationalizing experimental observations and resolving earlier discrepancies between theory and experiment.

## Abstract (web summary)

Competing charge density wave states were recently reported in the bilayer Kagome metal ScV_6_Sn_6_, but the mechanism remained unclear. Here the authors present a microscopic theory, highlighting the crucial role of anharmonic phonon-phonon interactions, and reconcile previous experimental and theoretical reports.

## Introduction

Kagome materials have emerged as promising platforms to study novel quantum phases of matter that arise from the interplay between lattice geometry, band topology, and electronic correlations^1–3^. Typical electronic band structures of kagome materials include Dirac points, flat bands, and van Hove singularities, serving as rich sources for a variety of structural and electronic instabilities^4–8^. Indeed, exotic electronic states such as superconductivity^9–11^, charge density waves (CDWs)^9,12–15^, pair density waves^16^, non-trivial topological states^9,17^, and electronic nematicity^18^, have been observed in representative kagome metals *A*V_3_Sb_5_ (*A*=K, Rb, and Cs)^19^. Of these, the CDW state exhibits unconventional properties including time-reversal and rotational symmetry breaking^12,18,20–26^, and an unconventional interplay with superconductivity featuring a double superconducting dome^27–30^. This has sparked remarkable interest and controversy, prompting the exploration of other material candidates in the quest for a comprehensive understanding of the CDW state in kagome materials.

In this context, the newly discovered bilayer kagome metals *R*V_6_Sn_6_ (*R* is a rare-earth element) have attracted much attention^31–51^. Among the *R*V_6_Sn_6_ series, the non-magnetic ScV_6_Sn_6_ compound is the only one that has been reported to undergo a CDW transition, which occurs below *T*_CDW_ ≈ 92 K^38^. The wave vector ${{{{\bf{q}}}}}_{3}=(\frac{1}{3},\frac{1}{3},\frac{1}{3})$, often described as corresponding to a $\sqrt{3}\times \sqrt{3}\times 3$ periodicity in real space, has been identified as the ordering vector of the CDW state through x-ray diffraction (XRD)^38^, neutron diffraction^38^, and inelastic x-ray scattering (IXS)^39,40^. The in-plane $\sqrt{3}\times \sqrt{3}$ periodicity has been further confirmed using surface-sensitive techniques such as scanning tunneling microscopy^41,42^ and angle-resolved photoemission spectroscopy^42,43^. It has also been established that the CDW distortion is dominated by out-of-plane displacements of Sc and Sn atoms^38,47^, which exhibit strong electron-phonon coupling^39,40,42,48^. Interestingly, short-range order with ${{{{\bf{q}}}}}_{2}=(\frac{1}{3},\frac{1}{3},\frac{1}{2})$, corresponding to $\sqrt{3}\times \sqrt{3}\times 2$ periodicity, has been detected above *T*_CDW_, but it is supressed below *T*_CDW_ when the dominant **q**_3_ CDW develops^39,40,48^.

On the theoretical front, density functional theory (DFT) calculations have reproduced the competing CDW orders^47^, but all previous DFT studies have found that the **q**_2_ CDW order is the lowest energy ground state^39,47,52–54^, in stark contrast to experimental reports. Various mechanisms have been proposed to explain the observed **q**_3_ CDW ground state, including configurational entropy^52^, the order-by-disorder mechanism^53^, and large fluctuations from flat phonon soft modes^54^. These scenarios are based on harmonic phonon dispersions, which exhibit multiple imaginary phonon modes and a theory capable of quantitatively explaining the temperature-driven phase transition from the **q**_2_ order to the **q**_3_ CDW order is missing. Moreover, the inclusion of electronic temperature in DFT calculations is also insufficient to predict the observed CDW transition, which is overestimated by thousands of degrees. These discrepancies between theoretical models and experimental observations pose a key challenge to achieving a comprehensive understanding of CDW states in bilayer kagome metals.

In this work, we present a first principles theory of CDW order in ScV_6_Sn_6_ that explains the reported experimental observations and resolves earlier discrepancies between theory and experiment. Our theory is based on the inclusion of anharmonic phonon-phonon interactions, and captures the observed temperature dependence of charge orders, with a **q**_2_ distortion occurring at a higher temperature that is subsequently suppressed by the dominant low-temperature **q**_3_ charge ordering. Moreover, we calculate a phase diagram comparing the two charge orders as a function of the in-plane and out-of-plane lattice parameters, and suggest a clear pathway for using compressive bi-axial strain to experimentally stabilize a CDW with a dominant **q**_2_ wave vector in ScV_6_Sn_6_. We also predict that Ge or Pb doping at the Sn site can control CDW order, and propose ScV_6_Pb_6_ as another CDW material within the 166 kagome family. Finally, we rationalize discrepant earlier theoretical models by highlighting that the relative stability of the competing CDW states in ScV_6_Sn_6_ exhibits a complex dependence on the number of valence electrons included in the calculations and on the out-of-plane lattice parameter.

## Results and discussion

**Fig. 1:**
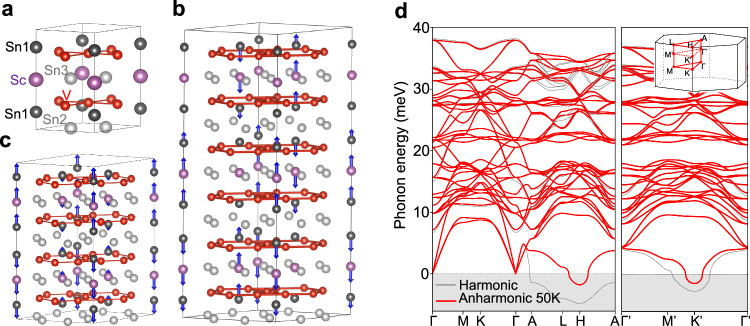
CDW instabilities in pristine ScV_6_Sn_6_ and corresponding CDW structures. **a** Crystal structure of pristine ScV_6_Sn_6_. **b**, **c** CDW displacement patterns (blue arrows) for the **b** **q**_3_ ($\sqrt{3}\times \sqrt{3}\times 3$) and **c** **q**_2_ ($\sqrt{3}\times \sqrt{3}\times 2$) orders. The arrows are calculated through the structural difference of the relaxed CDW structure and the pristine structure. **d** Harmonic (gray) and anharmonic (red) phonon dispersions of pristine ScV_6_Sn_6_. The inset shows the Brillouin zone of pristine ScV_6_Sn_6_ with certain high symmetry points labeled. The prime notation designates the high symmetry points on the ${q}_{z}=\frac{1}{3}$ plane.

The high temperature pristine phase of ScV_6_Sn_6_ crystallizes in the hexagonal space group *P*6/*mmm* (Fig. 1a). The primitive cell contains one Sc atom (Wyckoff position 1*a*), six equivalent V atoms (6*i*), and three pairs of nonequivalent Sn atoms, labeled as Sn1 (2*e*), Sn2 (2*d* ), and Sn3 (2*c*). The crystal structure consists of two kagome layers of V and Sn1 atoms, a triangular layer of Sc and Sn3 atoms, and a honeycomb layer of Sn2 atoms. In the two kagome layers, the Sn1 atoms buckle in opposite directions relative to each V kagome sublattice. The Sn1 and Sc atoms form a chain along the c axis. Notably, the smallest *R*-site ion radius of ScV_6_Sn_6_ among the *R*V_6_Sn_6_ series leads to the formation of Sn1-Sc-Sn1 trimers mediated by a shortening of the Sc-Sn1 bonds and an elongation of the Sn1-Sn1 bonds along the chain. This unique structural feature results in more space for the Sn1-Sc-Sn1 trimers to vibrate along the c direction, a characteristic absent in other *R*V_6_Sn_6_ compounds without a trimer formation, which has been demonstrated to be crucial to the formation of the CDW^44,48^. Motivated by this observation, the calculations reported below are performed using the PBEsol exchange-correlation functional^55^, which gives better agreement with the experimentally measured out-of-plane lattice parameter compared to the often-used PBE exchange-correlation functional^56^. Nonetheless, the overall conclusions of our work are independent of the exchange-correlation functional used (See Supplementary Note 2).

Figure 1d shows the calculated harmonic and anharmonic phonon dispersions of pristine ScV_6_Sn_6_. The harmonic phonon dispersion shows multiple dynamical instabilities, which appear as imaginary frequencies in the phonon dispersion (represented by the gray area in Fig. 1d). The harmonic instabilities span a wide region of the Brillouin zone, including the whole ${q}_{z}=\frac{1}{2}$ plane represented by the *A*-*L*-*H*-*A* closed path, and also including other values of *q*_*z*_, for example the ${K}^{{\prime} }$ point at the ${q}_{z}=\frac{1}{3}$ plane. Specifically, the calculated harmonic instabilities include the CDW instabilities reported experimentally with wave vectors **q**_3_ and **q**_2_, which correspond to the ${K}^{{\prime} }$ and *H* points of the Brillouin zone, respectively; but also include many other instabilities. Interestingly, when anharmonic phonon-phonon interactions^57–59^ are included, most harmonic instabilities disappear, leaving only two at the ${K}^{{\prime} }$ and *H* points at finite temperatures. This aligns with available experimental reports, which observe only those two instabilities.

Figure 1b, c illustrate the CDW structures optimized along the imaginary phonon modes at **q**_3_ and **q**_2_, respectively. The distortions of both CDW orders mainly occur along the Sn1-Sc chains, where the Sn1-Sc-Sn1 trimers are displaced to form  × 3 and  × 2 CDW periodicities along the c axis for the **q**_3_ and **q**_2_ orders, respectively. The **q**_3_ CDW order involves three trimers exhibiting a stationary-up-down pattern while the **q**_2_ CDW order involves two trimers with an up-down pattern. In both CDW orders, the trimers in one Sc1-Sn chain alternate their displacement along the c axis relative to the other Sc1-Sn chains, resulting in the $\sqrt{3}\times \sqrt{3}$ in-plane periodicity.

**Fig. 2:**
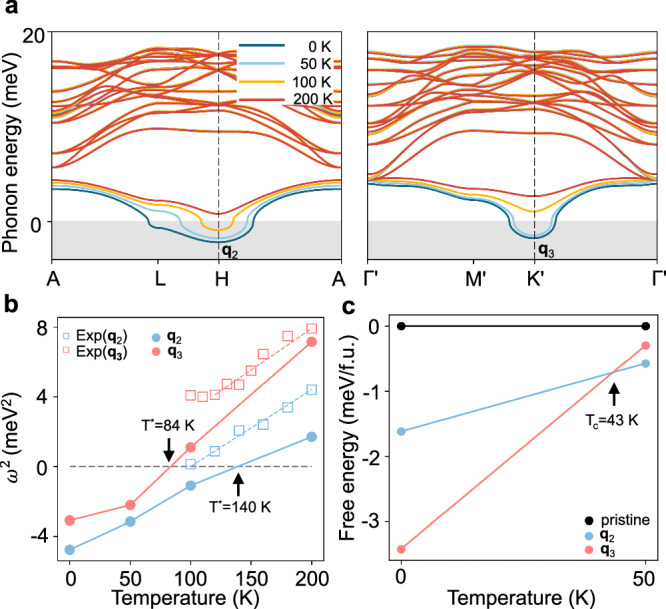
Anharmonic phonon dispersions and anharmonic free energy. **a** Anharmonic phonon dispersions at 0 K, 50 K, 100 K, and 200 K. **b** Squared phonon frequency *ω*^2^ of the lowest energy phonon modes at the H and ${K}^{{\prime} }$ points with respect to ionic temperature. The calculated anharmonic frequencies are shown in circles and the experimental results^40^ are represented by squares. **c** Helmholtz free energy of the two CDW structures compared to the pristine structure.

To further characterize the CDW of ScV_6_Sn_6_, we investigate the temperature dependence of phonon dispersions using anharmonic calculations within the stochastic self-consistent harmonic approximation^57–59^. Figure 2a displays the anharmonic phonon dispersion of pristine ScV_6_Sn_6_ at temperatures of 0 K, 50 K, 100 K and 200 K. The majority of phonon branches show a negligible temperature dependence, with the key exception of a pronounced softening of the lowest energy phonon branch at **q**_2_ (*H* point) and **q**_3_ (${K}^{{\prime} }$ point) upon cooling. The squared frequency *ω*^2^ of the soft modes should exhibit a linear behavior with respect to temperature near the vanishing point^60^, and we use a linear fit to extract the transition temperatures T^*^ associated with each phonon softening (Fig. 2b). At high temperature (200 K), the pristine ScV_6_Sn_6_ phase is dynamically stable and the phonon frequencies at both **q** vectors are real. Interestingly, the frequency at **q**_2_ becomes imaginary at a higher temperature of 140 K compared to the temperature of 84 K at which the frequency at **q**_3_ becomes imaginary. This implies that, starting from the high temperature pristine phase, the first distortion to develop with decreasing temperature is that associated with **q**_2_, rationalizing the short-range **q**_2_ order above the **q**_3_ CDW transition temperature observed in XRD^48^ and IXS^40^.

Our work clearly identifies anharmonic phonon-phonon interaction as the driving mechanism underlying the observed temperature-induced CDW transition in ScV_6_Sn_6_. In this context, our anharmonic phonon results exhibit remarkable quantitative agreement with experimental data (Fig. 2b). This should be contrasted with the significant overestimation of temperature scales (with critical temperatures of 2000 K and 5500 K) obtained by adjusting electronic temperature only via changing smearing values in harmonic phonon calculations (See Supplementary Note 3).

Comparing the calculated Helmholtz free energy between the **q**_2_ and **q**_3_ CDW orders, we observe a crossover from **q**_2_ to **q**_3_ CDWs (Fig. 2c). The **q**_2_ CDW order is more stable at high temperature and the free energy difference decreases as the temperature decreases. The crossover occurs approximately at *T*_*c*_ = 43 K, which is lower than the onset of the **q**_3_ CDW instability T^*^ = 84 *K* in Fig. 2b. The predicted transition temperature *T*_*c*_ of the **q**_3_ CDW (≈ 43 K) is lower than experimental values of 92 K^61^ and 84 K^48^. This quantitative discrepancy is attributed to the sensitivity of *T*_*c*_ on the volume of the system, as discussed in detail below, and more generally to the inherent temperature-independent limitations of DFT calculations, such as those arising from the choice of exchange-correlation functional. At 0 K, the **q**_3_ CDW structure is more stable by 1.80 meV/f.u. compared to the **q**_2_ CDW structure.

**Fig. 3:**
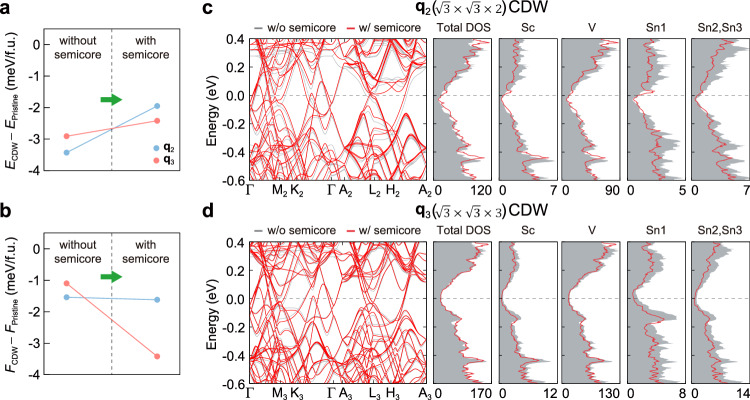
Effect of semi-core states and anharmonic quantum effects on the energetics between CDW states. **a** Total electronic energy and (**b**) free energy of CDW states relative to the pristine state at 0 K with and without including semi-core states as valence states. The free energy contains total electronic energy and phonon energy that arises from both harmonic and anharmonic quantum effects. **c**, **d** Electronic band structures and density of states (DOS) calculated with and without treating semi-core states as valence states for (**c**) the **q**_2_ and **d** the **q**_3_ CDW states. The semi-core states refer to the 3*s* and 3*p* orbitals of Sc atoms and the 4*d* orbitals of Sn atoms.

Our DFT calculations correctly predict the **q**_3_ CDW to be the lowest energy ground state, consistent with experiments^38–40,48^. Puzzlingly, all earlier DFT studies^47,52,53^ had predicted the **q**_2_ distortion to be the ground state, in stark contrast to the experimental reports and to our results. To rationalize this, we highlight that in our calculations we have established that the inclusion of electronic semi-core states in the valence is necessary to stabilize the **q**_3_ CDW order (Fig. 3). Using a standard pseudopotential with valence electrons 3*d*^1^4*s*^2^ for Sc atoms and 5*s*^2^5*p*^2^ for Sn atoms, which excludes the semi-core states from the valence, the total electronic energy of the **q**_2_ CDW is lower than that of the **q**_3_ CDW by 0.52 meV/f.u. (Fig. 3a), a conclusion reached by previous DFT calculations^47,52–54^. The **q**_2_ CDW remains the ground state in the free energy, stabilized by 0.44 meV/f.u. over the **q**_3_ CDW (Fig. 3b). The free energy includes both total electronic energy and phonon energy, with phonon contributions encompassing both harmonic and anharmonic zero-point energy at 0 K. By contrast, a pseudopotential that includes the *s* and *p* semi-core states in Sc atoms and the *d* semi-core states in Sn atoms as valence states, predicts the **q**_3_ CDW to be the ground state, regardless of phonon contributions. The **q**_3_ CDW structure is further stabilized upon considerinng phonon contributions, increasing the energy difference between the **q**_2_ and **q**_3_ CDWs from 0.47 to 1.80 meV/f.u. (Fig. 3a, b).

To gain further insight into the role of the pseudopotential, we analyse the effect of semi-core states on the electronic structures of the **q**_2_ and **q**_3_ distorted phases (Fig. 3c, d). We find that including semi-core states shifts the overall band dispersions and DOS of the occupied states upward, leading to a reduced total energy gain of both CDW states upon formation from the pristine structure (Fig. 3a). Specifically, the total energy gain of the CDW structures over the pristine structure decreases from  −3.43 to  −1.95 meV/f.u. for the **q**_2_ CDW, and from  −2.91 to  −2.42 meV/f.u. for the **q**_3_ CDW. The larger reduction in total energy gain for the **q**_2_ CDW is attributed to more significant changes in its electronic structure arising from larger structural changes in the **q**_2_ CDW, particularly in the bond lengths in the Sn1-Sc-Sn1 chains. These bond lengths change by up to 0.065 Å in the **q**_2_ CDW, compared to a maximum change of 0.022 Å in the **q**_3_ CDW upon the inclusion of semi-core states (see Supplementary Table S4 for details). This demonstrates that semi-core effects have a more pronounced impact on the **q**_2_ CDW than on the **q**_3_ CDW. Furthermore, unlike in the **q**_3_ CDW state, we observe that semi-core states affect the electronic structure near the Fermi level in the **q**_2_ CDW. The atom-projected DOS shows that the Sc and Sn1 states, which are responsible for the CDW state, are particularly influenced, suggesting that the corrections in the Sn1-Sc-Sn1 chains alter the Fermi surface and associated properties.

The validity of our theoretical calculations is confirmed by cross-checking with pseudopotential-free all-electron wien2k calculations (see Supplementary Note 4). We find that the vasp results, obtained by including semi-core states as valence, show remarkable agreement with the wien2k results in terms of the total energy of both pristine and CDW structures, as well as their atomic and electronic structures. Overall, this demonstrates that the inclusion of semi-core electron states in the valence is necessary to obtain a theoretical model that correctly predicts the ground state CDW order of ScV_6_Sn_6_. This explains and resolves the outstanding discrepancy between theory and experiment, and provides a foundation for the predictive model of the CDW state in bilayer kagome ScV_6_Sn_6_ described above. Additionally, we note that the calculated energy difference between the two CDWs is small (less than 2 meV/f.u.), highlighting the competing nature of the two CDWs in ScV_6_Sn_6_. This suggests that the competition between the two CDW orders can be easily manipulated via external perturbations, as discussed in detail below.

**Fig. 4:**
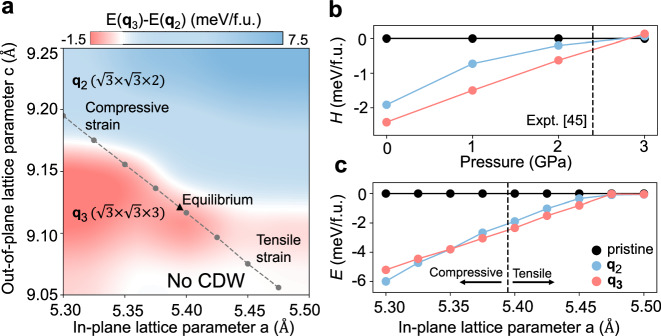
Manipulation of competing CDWs in ScV_6_Sn_6_ via strain and pressure. **a** Phase diagram of the competing **q**_2_ and **q**_3_ CDW orders in the in-plane and out-of-plane lattice parameter space. Color bar represents the total energy difference between the **q**_2_ and **q**_3_ structures. The gray dashed line shows the change of lattice parameters under in-plane biaxial strain. **b** Relative enthalpy *H* = *E* + *PV* of the **q**_2_ and **q**_3_ CDWs to pristine structure. Vertical dashed line indicates the experimental value of critical pressure of 2.4 GPa that CDW orders are totally suppressed^45^. **c** Total energy of the **q**_2_ and **q**_3_ CDWs as a function of in-plane biaxial strain.

Having established the correct theory of the competing CDWs in ScV_6_Sn_6_, we explore the phase diagram in lattice parameter space which provides important insights for manipulating the competing CDWs. Figure 4a shows a phase diagram of the **q**_2_ and the **q**_3_ CDW orders as a function of the in-plane and out-of-plane lattice parameters. We find that the out-of-plane lattice parameter holds the key to control competing the CDW orders. This can be rationalized by noting that the out-of-plane lattice parameter determines the space available for the distortions of adjacent Sn1-Sc-Sn1 trimers and thus dominates the energetics between competing CDWs. A smaller out-of-plane lattice parameter restricts the available space for Sn1-Sc-Sn1 trimers to distort, favoring an additional stationary configuration of trimers in **q**_3_ CDW. Conversely, a larger out-of-plane lattice parameter facilitates the collective distortions of trimers and favors the **q**_2_ CDW.

In addition, we discuss the effects of the hydrostatic pressure and in-plane biaxial strain on the competing CDWs. Hydrostatic pressure suppresses both CDW orders (Fig. 4b), as increasing pressure decreases the out-of-plane lattice parameter and thus the space available for Sn1-Sc-Sn1 trimers to distort. Our theoretical value for the critical pressure (≈ 2.8 GPa) at which the **q**_3_ CDW disappears is in remarkable agreement with the experimental value of 2.4 GPa^45^. Similary, in-plane biaxial tensile strain leads to a decrease in the out-of-plane lattice parameter, suppressing both CDW orders (Fig. 4c). By contrast, in-plane compressive strain increases the out-of-plane lattice parameter, enhancing the stability of both CDW orders. Interestingly, the **q**_2_ CDW order becomes the ground state under the compressive strain. The predicted value of critical compressive strain is just around 1%, which should be experimentally accessible. These predictions warrant further experimental studies to corroborate our findings.

**Fig. 5:**
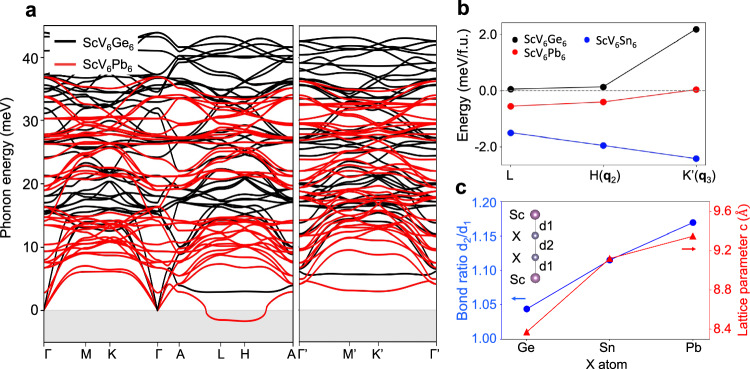
Control of CDW instabilities in ScV_6_Sn_6_ via doping. **a** Harmonic phonon dispersions of ScV_6_Ge_6_ and ScV_6_Pb_6_. **b** Total energy of various CDW structures relative to the pristine structure in ScV_6_X_6_ materials (X=Ge, Sn, and Pb). **c** Bond ratio *d*_2_/*d*_1_ in X-Sc-X chains and out-of-plane lattice parameter in the pristine structure of ScV_6_X_6_ materials (X=Ge, Sn, and Pb).

Finally, we propose the doping of Pb or Ge at the Sn site as a further strategy to control competing CDWs in ScV_6_Sn_6_ and to explore the microscopic mechanisms underlying CDW instabilities within the 166 bilayer kagome family. To investigate the effect of doping, we fully substitute the Sn atom with other Group 14 elements, Ge and Pb. Figure 5a displays the harmonic phonon dispersion of ScV_6_Pb_6_ (red lines) and ScV_6_Ge_6_ (black lines). The phonon dispersion of ScV_6_Pb_6_ shows imaginary modes at the *L* and *H* points, indicating CDW instabilities. The calculated total energy (Fig. 5b) confirms that ScV_6_Pb_6_ has a CDW ground state, with the *L* distortion slightly favored over the **q**_2_ distortion. We note that the **q**_3_ instability is not present in this compound. In contrast, ScV_6_Ge_6_ exhibits no imaginary modes, consistent with the calculated total energy showing that the CDW structure is unstable compared to the pristine structure. The bond ratio *d*_2_/*d*_1_ in X-Sc-X chains within the ScV_6_X_6_ compounds (X = Ge, Sn, and Pb) is an important indicator for the emergence of CDWs, with the ratio increasing as the atomic radius increases from Ge to Pb (Fig. 5c). A larger bond ratio is one of the necessary conditions for CDWs to emerge in this kagome system, as the CDWs displacement patterns consist of the vibration of X-Sc-X trimers. These vibrations are promoted by the larger bond difference between X-Sc and X-X atoms, which corresponds to a larger bond ratio. In the case of ScV_6_Sn_6_, the bond ratio is reduced to nearly 1 when Sc is substituted by larger atoms like Y or Gd, leading to the disappearance of CDWs^44^. Similarly, in ScV_6_Ge_6_, the bond ratio is close to 1, making trimerisation unfavorable and resulting in the absence of CDWs. Additionally, we find that there exists an optimal range of bond ratios that drive CDW formation, as the calculated energy gain of CDW states over the pristine structure diminishes in the Pb case compared to the Sn case, despite Pb having a larger bond ratio.

We note that the **q**_3_ CDW order is less stable compared to the pristine case in both the Ge and Pb compounds, and thus cannot be observed. In the Pb case, our theory predicts that the *L* and *H* (**q**_2_) CDWs are dominant, which we speculate is due to the larger out-of-plane lattice parameters (Fig. 5c), where we leave the detailed characterization as future work. Our results suggest that partial doping of Pb or Ge at the Sn site could suppress the **q**_3_ distortion, potentially leading to the dominance of the **q**_2_ CDW. We also predict ScV_6_Pb_6_ as a new CDW compound within the 166 bilayer kagome family. These predictions warrant further experimental studies to explore the role of doping in ScV_6_Sn_6_.

In conclusion, we have demonstrated the decisive role of temperature, captured by anharmonic phonon-phonon interactions, and volume, on the competition between the **q**_2_ and **q**_3_ charge orders in ScV_6_Sn_6_. Our work fully resolves the controversy between previous theoretical and experimental studies: a correct theoretical description of CDW order requires the inclusion of semi-core electron states as valence, and the use of a correct out-of-plane c lattice parameter. Our theory also elucidates the experimentally observed temperature-induced CDW phase transition from a high temperature **q**_2_ to a low temperature **q**_3_ order. Finally, we predict that in-plane biaxial strain can be used to manipulate the competing CDW orders, and propose that compressive strain could be used to experimentally discover a regime in which the **q**_2_ CDW dominates. We also suggest Ge or Pb doping at the Sn site can tune the competing CDW orders, and we predict ScV_6_Pb_6_ as a new CDW material, stimulating future experimental works. More generally, our findings contribute to the wider effort of understanding CDW states in kagome metals, including the prototypical CsV_3_Sb_5_^19^ and magnetic FeGe^62^, where the origin of the CDW states remains an open question^13,23,63–67^. Moreover, our work provides important insight into understanding and manipulating multiple CDW instabilities, such as those in CsV_3_Sb_5_ under doping and pressure^29,30^ as well as in the recently discovered room temperature CDW kagome metal LaRu_3_Si_2_^68,69^.

## Methods

### Electronic structure calculations

We perform density functional theory (DFT) calculations using the Vienna ab initio simulation package vasp^70,71^ implementing the projector-augmented wave method^72^. We use PAW pseudopotentials with valence configurations: 3*s*^2^3*p*^6^3*d*^1^4*s*^2^ (3*d*^1^4*s*^2^) for Sc atoms, 3*s*^2^3*p*^6^4*s*^2^3*d*^3^ (4*s*^2^3*d*^3^) for V atoms, and 5*s*^2^4*d*^10^5*p*^2^ (5*s*^2^5*p*^2^) for Sn atoms for the cases with (without) semi-core states. We approximate the exchange correlation functional with the generalized-gradient approximation PBEsol^55^ in the calculations reported in the main text. For comparison, we also perform calculations using the PBE^56^ exchange-correlation functional. We use a kinetic energy cutoff for the plane wave basis of 500 eV and a Methfessel-Paxton smearing of 0.02 eV. We use a Γ-centered **k**-point grid of size 15 × 15 × 8 for the primitive cell and commensurate **k**-point grids for the supercell calculations. All the structures are optimized until the forces are below 0.005 eV/Å. We perform a cross-check of the electronic structure calculations using the castep^73^ package (see Supplementary Note 1.2 for details), with norm-conversing pseudopotentials generated on-the-fly (NCP19), and employing identical valence configurations as those used in vasp calculations, including semi-core states. We also use the PBEsol^55^ exchange-correlation functional. We choose a kinetic energy cutoff for the plane wave basis of 1000 eV with a Gaussian smearing of 0.02 eV. We use a Monkhorst-Pack **k**-point grid with an applied half step shift if the number of **k**-points is even, which generates the exact same Gamma-centered **k**-point grid as that used in the vasp calculations. All the structures are optimized until the forces are below 0.005 eV/Å.

We also perform DFT calculations using the full-potential linearized augmented plane wave (FP-LAPW) method as implemented in the wien2k code^74^ using the PBEsol^55^ exchange-correlation functional. We use self-consistency cycle stopping criteria of 1 × 10^−6^ Ry for the energy and 1 × 10^−4^ e for the charge. The radii (R) of the muffin-tin (MT) spheres are taken to be 2.40, 2.26, and 2.38 Bohr for Sc, V, and Sn atoms, respectively. R_MT_ × K_max_ is set to 8.5 (confirming that using 9 yields the same results), where K_max_ is the cutoff value of the modulus of the reciprocal lattice vectors and R_MT_ is the smallest MT radius. The cutoff energy separating core and valence states is set to  −136 eV, with the valence electrons treated as 3*s*^2^3*p*^6^4*s*^2^3*d*^1^ for Sc atoms, 3*s*^2^3*p*^6^4*s*^2^3*d*^3^ for V atoms, and 4*s*^2^4*p*^6^4*d*^10^5*s*^2^5*p*^2^ for Sn atoms. All the structures are optimized until the forces are below 0.5 mRy/Bohr.

### Harmonic phonon calculations

We perform harmonic phonon calculations using the finite displacement method in conjunction with nondiagonal supercells^75,76^. The dynamical matrices are calculated on a Farey nonuniform **q** grid^77^ of size (3 × 3 × 2) ∪ (3 ×  3 × 3), which is commensurate with both **q**_2_ and **q**_3_. The final dynamical matrix is calculated through the force constant matrix on a target uniform **q** grid of size 3 × 3 × 6.

### Anharmonic phonon calculations

The anharmonic phonon calculations are performed using the stochastic self-consistent harmonic approximation (SSCHA)^57–59^, which is a non-perturbation method taking into account anharmonic effects at both zero and finite temperature. The free energy of the real system is variationally minimized with respect to an auxiliary harmonic system. This is done using stochastic importance sampling, in which the total energy, forces, and stresses for an ensemble of configurations of the auxiliary harmonic system are calculated using vasp. The associated electronic structure calculations are performed using a kinetic energy cutoff 300 eV, and we consider configurations commensurate with a 3 × 3 × 2 supercell and a 3 × 3 × 3 supercell. The number of configurations needed to converge the free energy Hessian is of the order of 1000. A Farey nonuniform **q** grid of size (3 × 3 × 2) ∪ (3 × 3 × 3) is used to get commensurate phonon results at both **q**_2_ and **q**_3_ (see Supplementary Note 1.1 for details). To get better prediction of the CDW transition temperature, the lattice parameters are fixed to the experimental values^38^. To obtain a more accurate energy comparison, the free energy calculations are performed using the same parameters as the electronic structure calculations.

## Supplementary information

Supplementary Information

Transparent Peer Review file

## Data Availability

The data that support the findings of this study are available within the paper and Supplementary Information. All other relevant data are available from the corresponding authors upon request.

## Code availability

The vasp code used in this study is a commercial electronic structure modeling software, available from https://www.vasp.at. The Quantum Espresso code used in this research is open source: https://www.quantum-espresso.org/. The castep code used in this study is freely available at https://www.castep.org/for academic research. The Wien2k code used in this study is a commercial software, available from http://susi.theochem.tuwien.ac.at/.
