# Supplementary material for: Origin of competing charge density waves in kagome metal ScV6Sn6
Source: Nat Commun. 2024 Nov 30;15:10428. doi: 10.1038/s41467-024-54702-3 (PMC11608277; doi:10.1038/s41467-024-54702-3)
Supplement: Supplementary file 1 — Supplementary Information [file 41467_2024_54702_MOESM1_ESM.pdf]

Supplementary Information for "Origin of competing charge density waves in kagome metal ScV_6Sn_6"

Kang Wang,[1] Siyu Chen,[1,2] Sun-Woo Kim,[1,*] and Bartomeu Monserrat[1,2,†]

[1] *Department of Materials Science and Metallurgy, University of Cambridge, 27 Charles Babbage Road, Cambridge CB3 0FS, United Kingdom*

[2] *Cavendish Laboratory, University of Cambridge, J. J. Thomson Avenue, Cambridge CB3 0HE, United Kingdom*

Contents

Supplementary Note 1. Convergence tests 2

1.1. Convergence of phonon dispersions 2

1.2. Cross-check with CASTEP 4

Supplementary Note 2. Comparison between PBE and PBEsol functionals 5

2.1. Lattice parameters 5

2.2. Harmonic phonon dispersions 5

Supplementary Note 3. Electronic temperature effects 7

Supplementary Note 4. Justification of the VASP pseudopotential 8

4.1. Total energy landscape of the pristine structure 8

4.2. Total energy gain of CDW states compared to the pristine structure 9

4.3. Bond lengths in the Sn1-Sc-Sn1 chains of the pristine and CDW structures 9

4.4. Electronic structures 11

Supplementary Note 5. Comparison with previous results 15

References 17

Supplementary Note 1. CONVERGENCE TESTS

1.1. Convergence of phonon dispersions

In this section, we investigate the convergence of phonon dispersions with respect to the $\mathbf{q}$-point grid size. Since the two competing CDW orders with wave vectors $\mathbf{q}_2$ and $\mathbf{q}_3$ correspond to the H and K$'$ points of the Brillouin zone, respectively, it is crucial to calculate the dynamical matrices directly at both H and K$'$ points to obtain reliable results. In general, calculated phonon frequencies are exact at any $\mathbf{q}$-point commensurate with the grid used. Therefore, the phonon frequencies at the H point $(\frac{1}{3}, \frac{1}{3}, \frac{1}{2})$ are exact for the uniform $3 \times 3 \times 2$ $\mathbf{q}$-point grid, and the phonon frequencies at the K$'$ point $(\frac{1}{3}, \frac{1}{3}, \frac{1}{3})$ are exact for the uniform $3 \times 3 \times 3$ grid. Using uniform grids, they are both *simultaneously* exact for the uniform $3 \times 3 \times 6$ grid. Instead, we can reach the same results using a non-uniform Farey grid [1] of size $(3 \times 3 \times 2) \cup (3 \times 3 \times 3)$ that leads to frequencies that are also *simultaneously* exact for both H and K$'$ points, but it is computationally more efficient than the uniform grid of size $3 \times 3 \times 6$. We confirm that the harmonic phonon dispersions obtained using the non-uniform Farey grid of size $(3 \times 3 \times 2) \cup (3 \times 3 \times 3)$ are the same with those obtained using the uniform

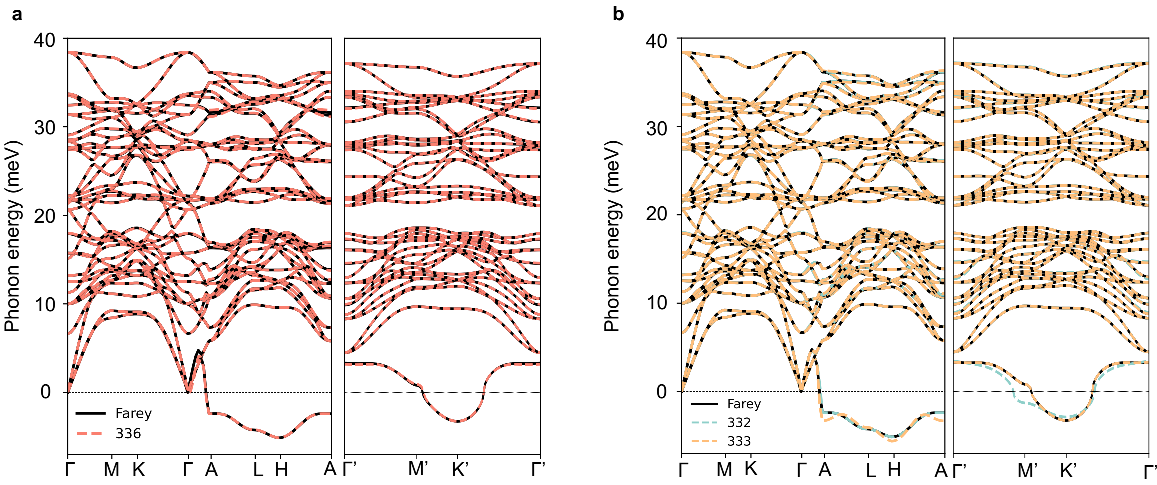

Supplementary Figure 1: **a** Calculated harmonic phonon dispersions using $\mathbf{q}$-point grid sizes of a $3 \times 3 \times 6$ uniform grid (red dashed line) and a $(3 \times 3 \times 2) \cup (3 \times 3 \times 3)$ non-uniform Farey grid (black solid line). **b** Calculated harmonic phonon dispersions using $\mathbf{q}$-point grid sizes of $3 \times 3 \times 2$ (cyan dashed line) and $3 \times 3 \times 3$ (yellow dashed line) uniform grids as well as a $(3 \times 3 \times 2) \cup (3 \times 3 \times 3)$ non-uniform Farey grid (black solid line).

$3 \times 3 \times 6$ grid (Supplementary Fig. 1).

We also apply the $(3 \times 3 \times 2) \cup (3 \times 3 \times 3)$ Farey grid to obtain anharmonic phonon dispersions, as calculating both H and K$'$ simultaneously at the anharmonic level using a uniform $3 \times 3 \times 6$ grid is computationally prohibitive. Moreover, we find that the Farey grid produces more substantial corrections in anharmonic calculations, as the phonon frequencies at the H and K$'$ points differ significantly depending on the **q**-point grid used (Supplementary Fig. 2). When using a $3 \times 3 \times 2$ **q**-point grid (blue solid lines), the phonon frequencies at K$'$, obtained through Fourier interpolation, are higher than those computed with the more accurate Farey grid (black dashed lines), resulting in the absence of the $\mathbf{q}_3$ CDW order even at $T = 50$ K. Similarly, with the $3 \times 3 \times 3$ **q**-point grid (red solid lines), the interpolated phonon frequencies at H are significantly lower than those from the Farey grid, leading to

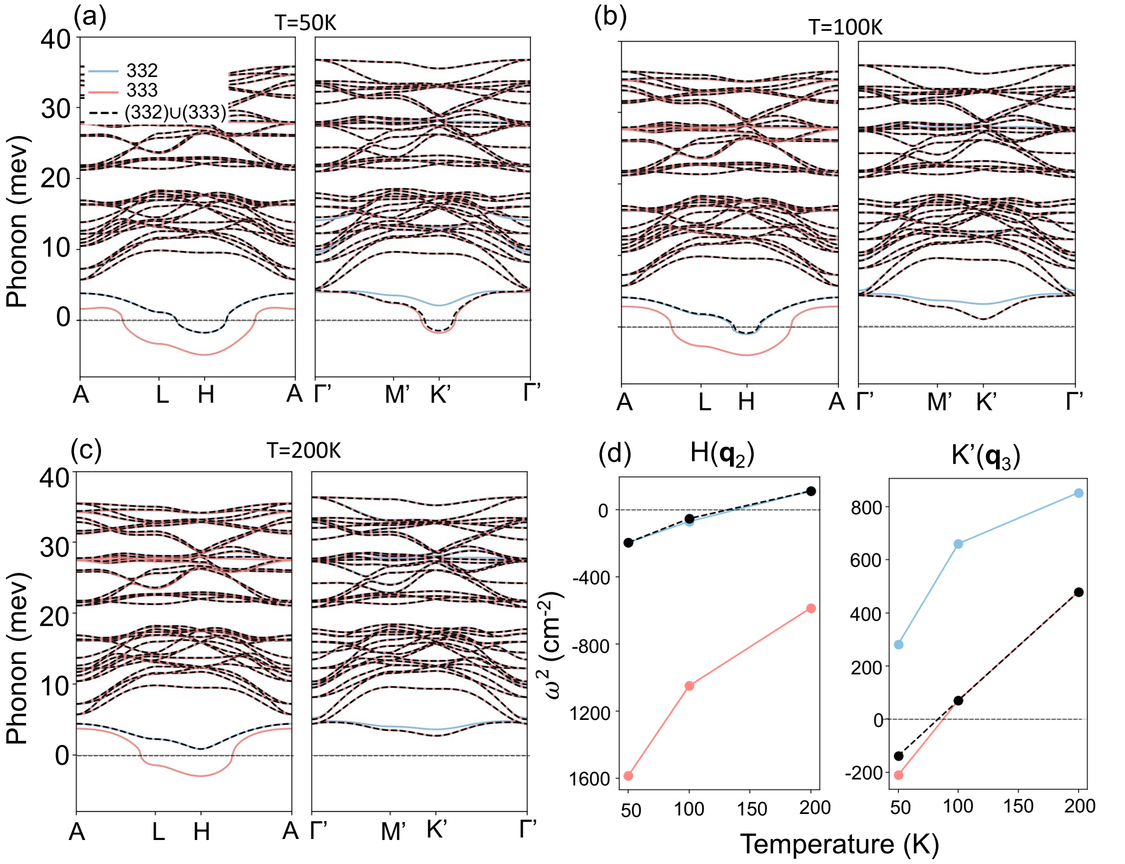

Supplementary Figure 2: Calculated anharmonic phonon dispersions using **q**-point grid sizes of $3 \times 3 \times 2$, $3 \times 3 \times 3$, and $(3 \times 3 \times 2) \cup (3 \times 3 \times 3)$ at (a) 50 K (b) 100 K and (c) 200 K. (d) The square of the lowest phonon frequency (ω^2) at the H ($\mathbf{q}_2$) and K$'$ ($\mathbf{q}_3$) points.

imaginary frequencies even at $T = 200\,\text{K}$. These artefacts from Fourier interpolation can only be correct by explicitly including both H and K$'$ points, as done with the Farey grid (black dashed lines), and these are the results presented in the main text.

1.2. Cross-check with CASTEP

To cross-check the results obtained from VASP, we perform calculations using CASTEP on the two CDW structures with the same valence configurations including semi-core states. The results from both VASP and CASTEP consistently show that the $\mathbf{q}_3$ CDW order is stable over the $\mathbf{q}_2$ CDW (Table 1). Specifically, the energy difference between the two CDW structures is 0.5 meV/f.u. and 1.1 meV/f.u. using VASP and CASTEP, respectively.

Supplementary Table 1: The total energy (meV/f.u.) of fully relaxed CDW structures compared to pristine structure.

	$\mathbf{q}_2$ CDW	$\mathbf{q}_3$ CDW	$E(\mathbf{q}_2) - E(\mathbf{q}_3)$
VASP	-1.95	-2.42	0.47
CASTEP	-1.54	-2.66	1.12

Supplementary Note 2. COMPARISON BETWEEN PBE AND PBESOL FUNCTIONALS

2.1. Lattice parameters

In the main text, we present the results obtained using the PBEsol exchange-correlation functional, as it yields a better agreement with the experimentally measured out-of-plane lattice parameter, a quantity that is crucial for CDW formation. Specifically, PBEsol gives an out-of-plane lattice parameter of $c = 9.12$ Å, which is closer to the experimental value of $c = 9.16$ Å compared to the PBE value of $c = 9.25$ Å (Table 2).

Supplementary Table 2: Lattice parameters of pristine ScV_6Sn_6 obtained using PBE and PBEsol.

Lattice parameter	PBE	PBEsol	Expt. [2]
In-plane a (Å)	5.46	5.39	5.47
Out-of-plane c (Å)	9.25	9.12	9.16

2.2. Harmonic phonon dispersions

Figure 3 shows the calculated phonon dispersions using PBEsol and PBE. The overall phonon dispersions look similar, including the imaginary branches. Both PBEsol and PBE show phonon instabilities at the H and K$'$ points, and the lowest imaginary frequency is observed at the H point.

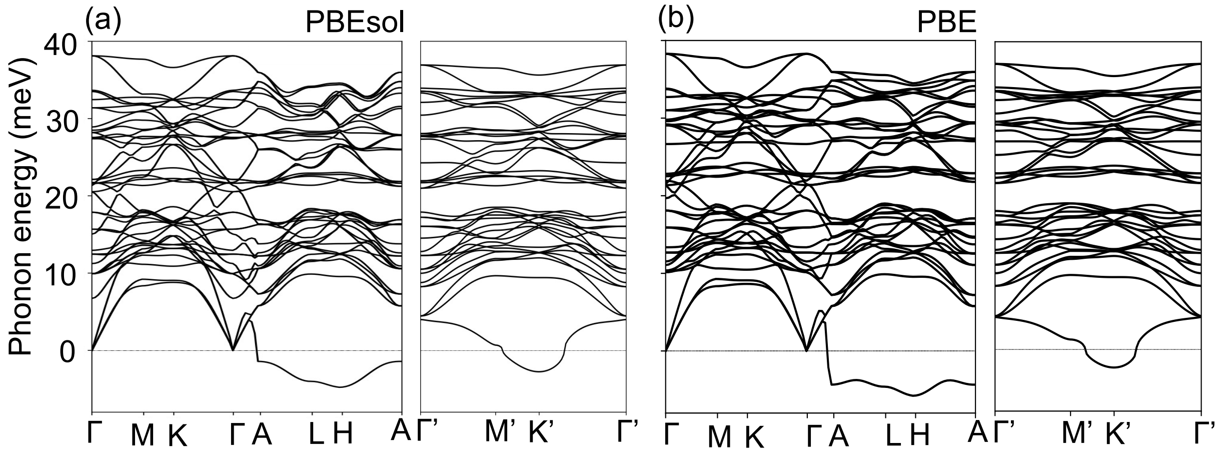

Supplementary Figure 3: The harmonic phonon dispersions obtained using the (a) PBEsol and (b) PBE functionals. A Farey grid of size $(3 \times 3 \times 2) \cup (3 \times 3 \times 3)$ is used to obtain the phonon dispersions.

Supplementary Note 3. ELECTRONIC TEMPERATURE EFFECTS

In this section, we investigate the effects of electron temperature on harmonic phonon dispersions. We calculate harmonic phonon dispersions over a range of smearing values from 0.02 eV to 0.5 eV, corresponding approximately to temperatures ranging from 300 K to 5,800 K [Supplementary Fig. 4(a)]. The phonon dispersions undergo changes with increasing electronic temperature, notably in the imaginary branches. The estimated transition temperatures for the $\mathbf{q}_2$ and $\mathbf{q}_3$ CDWs are 5500 K and 2000 K, respectively [Supplementary Fig. 4(b)].

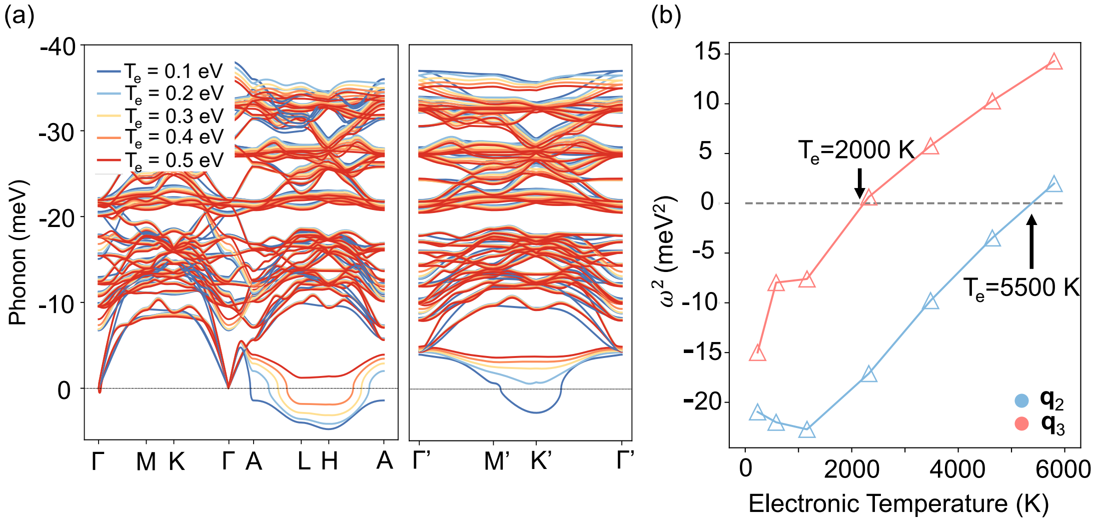

Supplementary Figure 4: (a) The calculated harmonic phonon dispersion using various electronic temperature. (b) Squared phonon frequency ω^2 of the lowest energy phonon modes at the H and K′ points with respect to the electronic temperature. Note that 0.1 eV is 1160.6 K.

Supplementary Note 4. JUSTIFICATION OF THE VASP PSEUDOPOTENTIAL

In this section, we justify our choice of the semi-core pseudopotential by comparing results with those from pseudopotential-free all-electron WIEN2K calculations. Below, we demonstrate the remarkable agreement between the VASP results using the pseudopotential with semi-core states and the WIEN2K results in terms of: (i) the total energy landscape of the pristine structure as a function of lattice parameters, (ii) the total energy gain of CDW states compared to the pristine structure, (iii) bond lengths in the Sn1-Sc-Sn1 chains of the pristine and CDW structures, and (iv) electronic structures.

4.1. Total energy landscape of the pristine structure

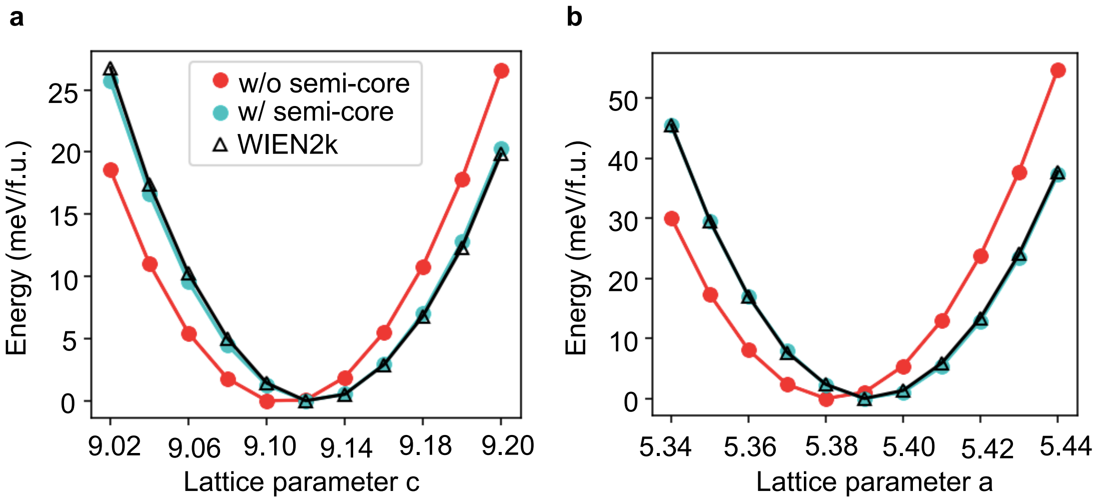

Supplementary Figure 5: Total energy of the pristine structure calculated using pseudopotential methods with and without semi-core states compared to all-electron WIEN2K results as a function of **a** out-of-plane lattice parameter c and **b** in-plane lattice parameter a.

Figure 5 shows the total energy of the pristine structure as a function of out-of-plane and in-plane lattice parameters, using pseudopotentials with and without semi-core states, compared to all-electron WIEN2K calculations. WIEN2K is frequently used as a high-accuracy reference to verify whether the pseudopotential accurately describes a system [3]. The data clearly demonstrate that the pseudopotential including semi-core states aligns

almost perfectly with WIEN2K results, indicating its superior accuracy compared to the pseudopotential without semi-core states.

4.2. Total energy gain of CDW states compared to the pristine structure

We find that the WIEN2K results closely align with those obtained using the pseudopotential that includes semi-core states. The WIEN2K calculations predict the $\mathbf{q}_3$ CDW as the ground state, which is consistent with the VASP results when semi-core states are included (Table 3). Crucially, without semi-core states, VASP fails to predict the correct ground state. Quantitatively, the total energy gain upon CDW formation is $-1.91\,\mathrm{meV/f.u}$ for the $\mathbf{q}_2$ CDW and $-2.69\,\mathrm{meV/f.u.}$ for the $\mathbf{q}_3$ CDW, resulting in an energy difference of $0.78\,\mathrm{meV/f.u.}$ between the two CDW states. This value is close to the VASP result with semi-core states, which is $0.47\,\mathrm{meV/f.u.}$ Notably, the total energy gain for the $\mathbf{q}_2$ CDW ($-1.91\,\mathrm{meV/f.u.}$) is very close to the value obtained with the pseudopotential including semi-core states ($-1.95\,\mathrm{meV/f.u.}$).

Supplementary Table 3: **Total energy of CDW states relative to the pristine structure.** In the WIEN2K calculations, the cutoff energy separating core and valence states is set to $-136\,\mathrm{eV}$, with the valence electrons treated as $3s^2 3p^6 4s^2 3d^1$ for Sc atoms, $3s^2 3p^6 4s^2 3d^3$ for V atoms, and $4s^2 4p^6 4d^{10} 5s^2 5p^2$ for Sn atoms.

Unit:meV/f.u.	VASP (w/o semicore)	VASP (w/ semicore)	WIEN2K
$\mathbf{q}_2$ CDW	-3.43	-1.95	-1.91
$\mathbf{q}_3$ CDW	-2.91	-2.42	-2.69

4.3. Bond lengths in the Sn1-Sc-Sn1 chains of the pristine and CDW structures

We first compare the bond lengths in the Sn1-Sc-Sn1 chains of the pristine structure. The pseudopotential without semi-core states yields $d_1 = 2.922\,\text{Å}$ and $d_2 = 3.274\,\text{Å}$ (Table 4;

bond lengths d_i are labelled in Supplementary Fig. 6), while the pseudopotential with semi-core states gives $d_1 = 2.933$ Å and $d_2 = 3.255$ Å. These latter values are closer to the WIEN2K and experimental results, demonstrating the improved accuracy provided by including semi-core states.

For the CDW structures, the bond lengths obtained using the pseudopotential with semi-core states again closely match those from WIEN2K, with a maximum difference of 0.024 Å compared to the WIEN2K results. In contrast, the bond lengths obtained without semi-core states show a larger maximum difference of 0.063 Å compared to the WIEN2K results. When comparing the VASP results with available experimental data for the $\mathbf{q}_3$ CDW structure, the results with semi-core states exhibit better agreement with the experiments, further validating the importance of including semi-core states for accurate predictions.

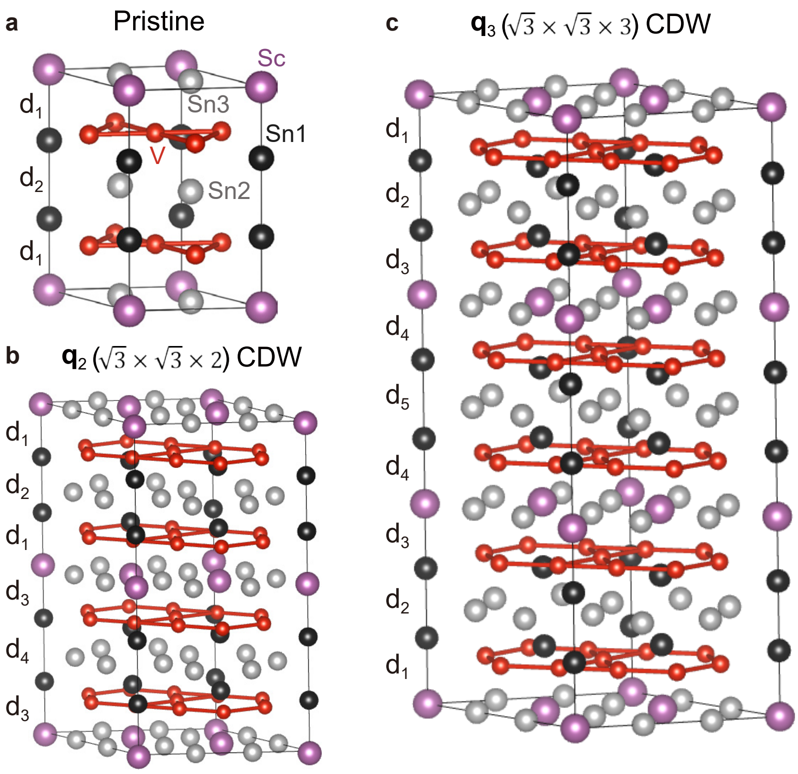

Supplementary Figure 6: Labelling of bonds in the Sn1-Sc-Sn1 chains of **a** the pristine, **b** the $\mathbf{q}_2$ CDW, and **c** the $\mathbf{q}_3$ CDW structures.

Supplementary Table 4: **Bond lengths (in Å) of Sn1-Sc-Sn1 chains.** The bond lengths d_i are labelled in Supplementary Fig. 6. In the WIEN2K calculations, the cutoff energy separating core and valence states was set to −136 eV, with the valence electrons treated as $3s^2 3p^6 4s^2 3d^1$ for Sc atoms, $3s^2 3p^6 4s^2 3d^3$ for V atoms, and $4s^2 4p^6 4d^{10} 5s^2 5p^2$ for Sn atoms. We use the fully relaxed lattice parameters employing PBEsol functional.

Structure	Bond	VASP (w/o semicore)	VASP (w/ semicore)	WIEN2K	Expt. [2]
Pristine	d_1	2.922	2.933	2.938	2.971
	d_2	3.274	3.255	3.245	3.218
$\mathbf{q}_2$ CDW	d_1	2.889	2.904	2.907	-
	d_2	3.044	3.063	3.052	-
	d_3	2.922	2.928	2.931	-
	d_4	3.608	3.543	3.545	-
$\mathbf{q}_3$ CDW	d_1	2.955	2.967	2.972	2.995
	d_2	3.148	3.126	3.106	3.074
	d_3	2.891	2.901	2.902	2.924
	d_4	2.917	2.925	2.927	2.949
	d_5	3.553	3.552	3.576	3.530

4.4. Electronic structures

As the CDW structures change with the inclusion of semi-core states, their electronic structures also change accordingly. We note that semi-core effects have a more pronounced impact on the $\mathbf{q}_2$ CDW than on the $\mathbf{q}_3$ CDW. This is evident from the larger changes in bond lengths in the Sn1-Sc-Sn1 chains for the $\mathbf{q}_2$ CDW (with a maximum change of 0.065 Å) compared to those in the $\mathbf{q}_3$ CDW (with a maximum change of 0.022 Å), as presented in Table 4. These bond length changes result in more significant alterations in the electronic structures of the $\mathbf{q}_2$ CDW, leading to more visible difference in the electronic structures upon including semi-core states, as shown in Supplementary Fig. 7 and Supplementary Fig. 8.

Again, the electronic structures obtained with semi-core states remarkably match those from WIEN2K for both CDW structures.

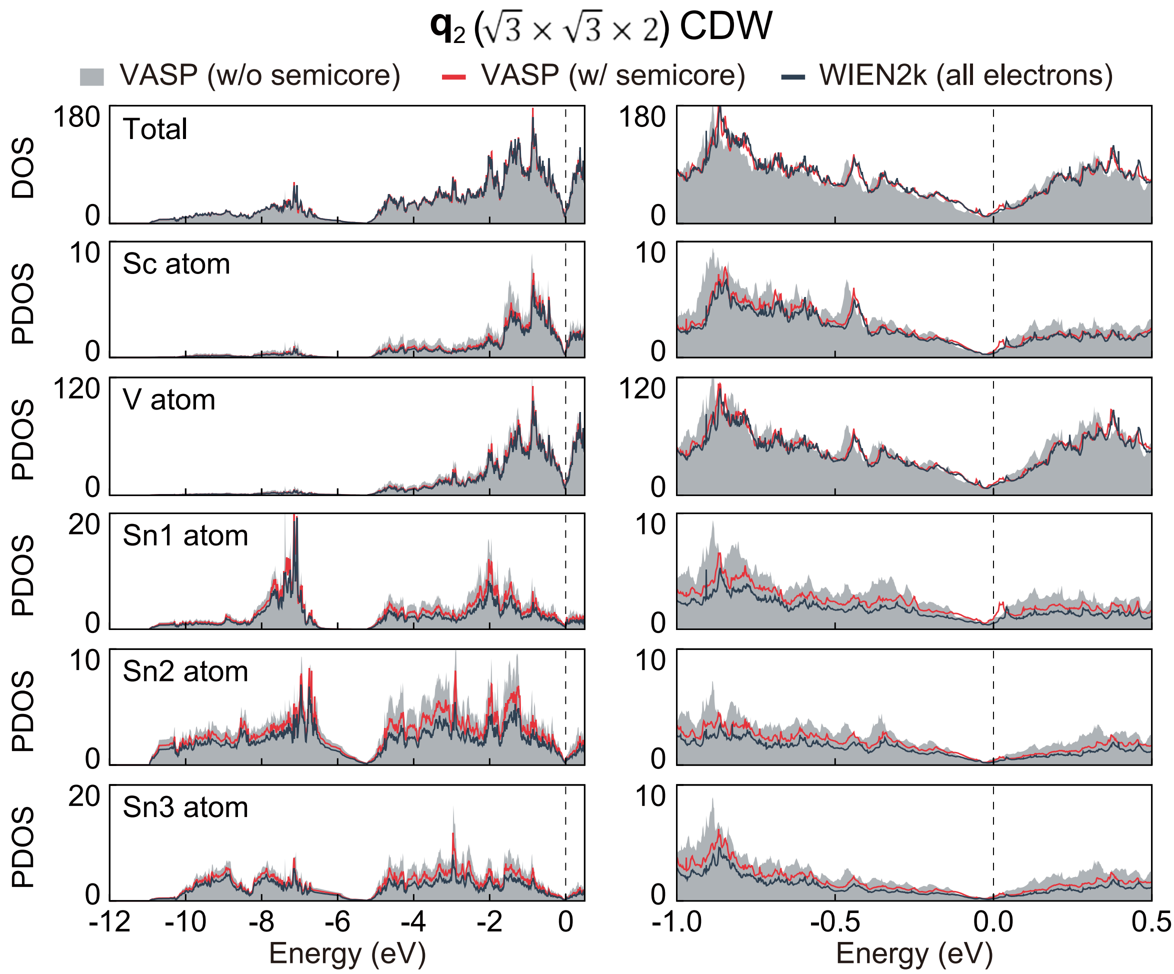

Supplementary Figure 7: Total density of states (DOS) and atom-projected density of states (PDOS) for the $\mathbf{q}_2$ $(\sqrt{3} \times \sqrt{3} \times 2)$ CDW calculated using VASP with and without semi-core states, compared with WIEN2K results.

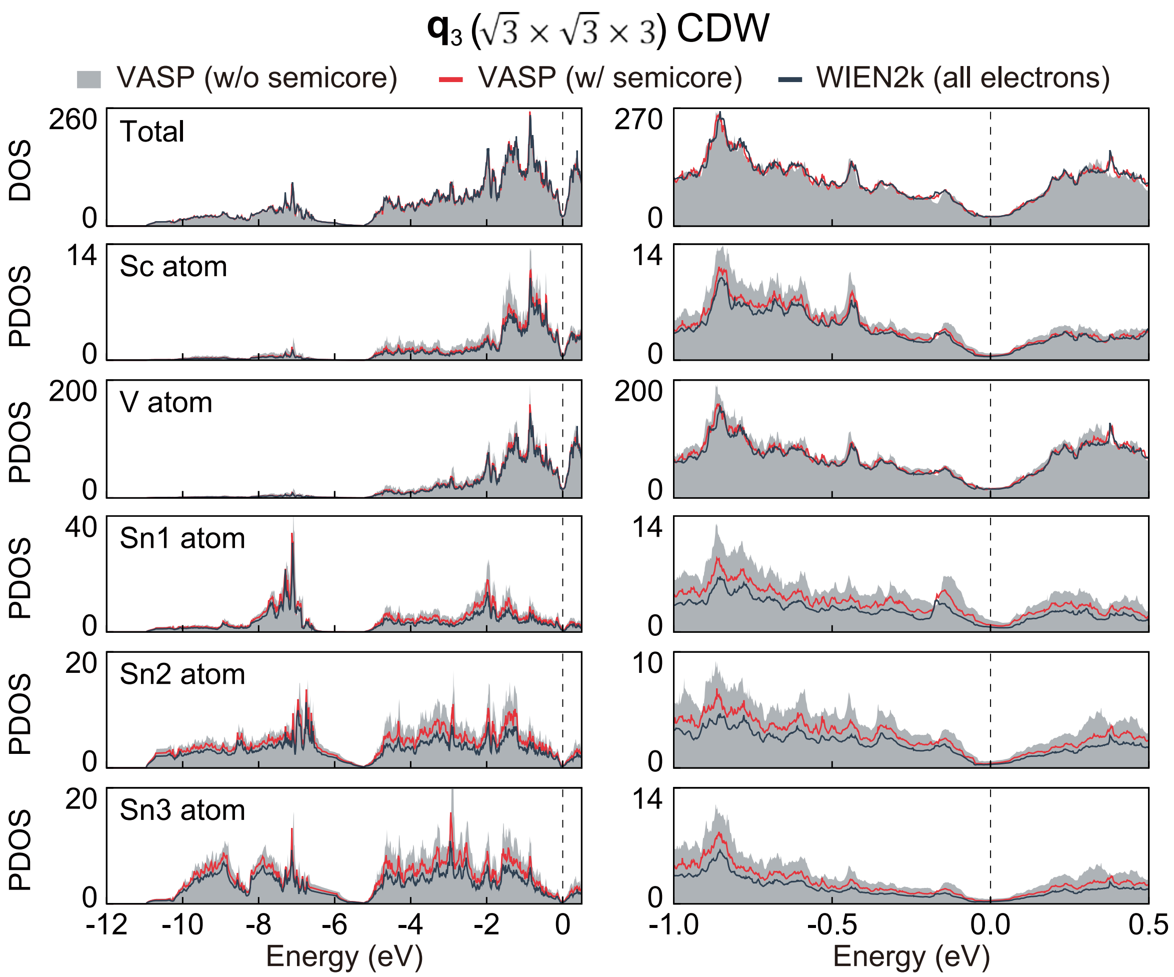

Supplementary Figure 8: Total density of states (DOS) and atom-projected density of states (PDOS) for the $\mathbf{q}_3$ $(\sqrt{3} \times \sqrt{3} \times 3)$ CDW calculated using VASP with and without semi-core states, compared with WIEN2K results.

We further present the inaccuracy of the pseudopotential without including semi-core states by demonstrating its failure to explain the experimental data. Figure 9 shows the phase diagram of the two CDW orders in lattice parameter spaces. In contrast to results with the semi-core states included, the pseudopotential without the semi-core states fails to predict the $\mathbf{q}_3$ CDW order across any of the lattice parameters explored.

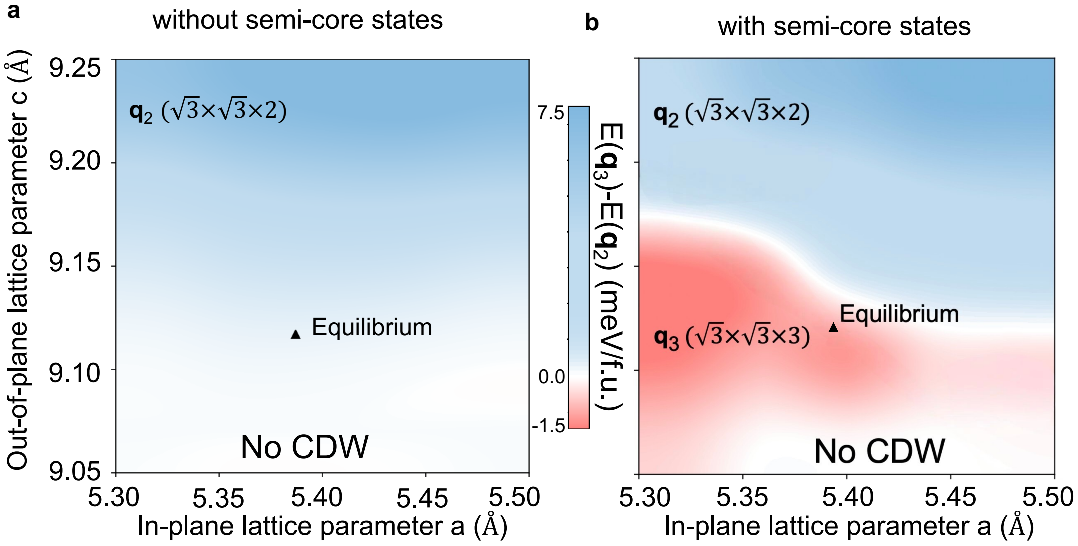

Supplementary Figure 9: Phase diagram of the competing $\mathbf{q}_2$ and $\mathbf{q}_3$ CDW orders in the in-plane and out-of-plane lattice parameter space, calculated using **a** without and **b** with semi-core states as valence states. Color bar represents the total energy difference between the $\mathbf{q}_2$ and $\mathbf{q}_3$ structures.

Supplementary Note 5. COMPARISON WITH PREVIOUS RESULTS

In this section, we calculate the energetics of the two competing CDW states as a function of the lattice parameter (Supplementary Fig. 10). This allows us to rationalize the previous reports and explain why they obtained a $\mathbf{q}_2$ CDW ground state rather than the experimentally observed $\mathbf{q}_3$ CDW ground state. The previous DFT calculations [4–8] were performed using the PBE functional, yielding an optimized out-of-plane lattice parameter of $c = 9.25$ Å, which is significantly larger than the experimental out-of-plane lattice parameter of $c = 9.16$ Å [?]. At the PBE-optimized lattice parameters, the $\mathbf{q}_2$ CDW is indeed calculated to be more stable than the $\mathbf{q}_3$ CDW (triangle in Supplementary Fig. 10). However, using the experimentally reported lattice parameters shows the $\mathbf{q}_3$ CDW is ground state (star in Supplementary Fig. 10). These results confirm the important role that the out-of-plane lattice parameter plays in determining the relative stability of the two CDW orders. The results also explain why previous DFT calculations failed to predict the $\mathbf{q}_3$ CDW ground state even if some of them considered the inclusion of the semi-core states in the valence states.

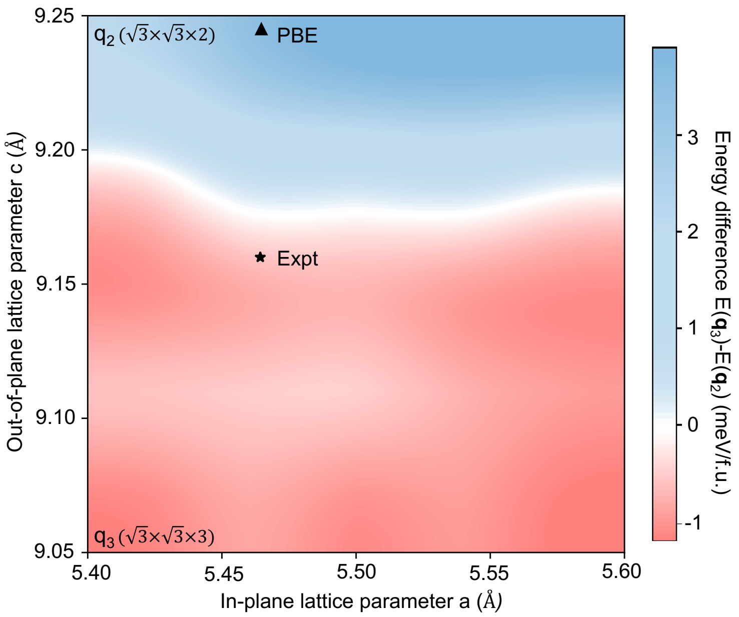

Supplementary Figure 10: Calculated phase diagram as a function of lattice parameters using the PBE functional. The color bar represents the energy difference between the $\mathbf{q}_2$ and $\mathbf{q}_3$ CDW structures. The triangle corresponds to the lattice parameters obtained using the PBE functional, while the star represents the lattice parameters reported experimentally [2].

[1] Chen, S., Salzbrenner, P. T. & Monserrat, B. Nonuniform grids for brillouin zone integration and interpolation. *Phys. Rev. B* **106**, 155102 (2022).

[2] Arachchige, H. W. S. *et al.* Charge Density Wave in Kagome Lattice Intermetallic ScV_6Sn_6. *Phys. Rev. Lett.* **129**, 216402 (2022).

[3] Lejaeghere, K. *et al.* Reproducibility in density functional theory calculations of solids. *Science* **351**, aad3000 (2016).

[4] Tan, H. & Yan, B. Abundant lattice instability in kagome metal ScV_6Sn_6. *Phys. Rev. Lett.* **130**, 266402 (2023).

[5] Liu, S. *et al.* Driving mechanism and dynamic fluctuations of charge density waves in the kagome metal SnV_6Sn_6. *Phys. Rev. B* **109**, L121103 (2024).

[6] Subedi, A. Order-by-disorder charge density wave condensation at $q = (\frac{1}{3}, \frac{1}{3}, \frac{1}{3})$ in kagome metal ScV_6Sn_6. *Phys. Rev. Mater.* **8**, 014006 (2024).

[7] Hu, H. *et al.* Kagome Materials I: SG 191, ScV_6Sn_6. Flat Phonon Soft Modes and Unconventional CDW Formation: Microscopic and Effective Theory. *arXiv preprint arXiv:2305.15469* (2023).

[8] Cao, S. *et al.* Competing charge-density wave instabilities in the kagome metal ScV_6Sn_6. *Nat. Commun.* **14**, 7671 (2023).
